# Co-Flocculation of Yeast Species, a New Mechanism to Govern Population Dynamics in Microbial Ecosystems

**DOI:** 10.1371/journal.pone.0136249

**Published:** 2015-08-28

**Authors:** Debra Rossouw, Bahareh Bagheri, Mathabatha Evodia Setati, Florian Franz Bauer

**Affiliations:** Institute for Wine Biotechnology, Department of Oenology and Viticulture, Private Bag X1, Stellenbosch University, Stellenbosch, 7600, South Africa; USDA Forest Service, UNITED STATES

## Abstract

Flocculation has primarily been studied as an important technological property of *Saccharomyces cerevisiae* yeast strains in fermentation processes such as brewing and winemaking. These studies have led to the identification of a group of closely related genes, referred to as the *FLO* gene family, which controls the flocculation phenotype. All naturally occurring *S*. *cerevisiae* strains assessed thus far possess at least four independent copies of structurally similar *FLO* genes, namely *FLO1*, *FLO5*, *FLO9* and *FLO10*. The genes appear to differ primarily by the degree of flocculation induced by their expression. However, the reason for the existence of a large family of very similar genes, all involved in the same phenotype, has remained unclear. In natural ecosystems, and in wine production, *S*. *cerevisiae* growth together and competes with a large number of other *Saccharomyces* and many more non-*Saccharomyces* yeast species. Our data show that many strains of such wine-related non-*Saccharomyces* species, some of which have recently attracted significant biotechnological interest as they contribute positively to fermentation and wine character, were able to flocculate efficiently. The data also show that both flocculent and non-flocculent *S*. *cerevisiae* strains formed mixed species flocs (a process hereafter referred to as co-flocculation) with some of these non-*Saccharomyces* yeasts. This ability of yeast strains to impact flocculation behaviour of other species in mixed inocula has not been described previously. Further investigation into the genetic regulation of co-flocculation revealed that different *FLO* genes impact differently on such adhesion phenotypes, favouring adhesion with some species while excluding other species from such mixed flocs. The data therefore strongly suggest that *FLO* genes govern the selective association of *S*. *cerevisiae* with specific species of non-*Saccharomyces* yeasts, and may therefore be drivers of ecosystem organisational patterns. Our data provide, for the first time, insights into the role of the *FLO* gene family beyond intraspecies cellular association, and suggest a wider evolutionary role for the *FLO* genes. Such a role would explain the evolutionary persistence of a large multigene family of genes with apparently similar function.

## Introduction

The ability of microbial cells to adhere to other cells or to substrates is an essential feature of important properties such as biofilm formation, invasive growth and sexual reproduction [1; 2]. These adhesion phenotypes are primarily dependent on properties of the cell wall, and can be adjusted as part of adaptive responses to environmental cues. In yeast, adhesion properties are primarily regulated in response to changes in environmental conditions such as nitrogen availability, glucose depletion, shortages of sterols and fatty acids, or changes in pH and ethanol levels [3; 4]. Flocculation is one such important adhesion-dependent phenotype, and has been used as a model to study the regulation of cell wall properties in *Saccharomyces cerevisiae*. Flocculating cells have the ability to adhere to one another to form large multicellular aggregates or ‘flocs’ which sediment to the bottom of the fermentation vessel [5; 6; 7; 8]. Various hypotheses regarding the role of flocculation have been proposed, in particular that floc formation may be a protective mechanism to resist environmental stresses, and may allow the generation of nutritionally rich micro-environments by selective lysis [9].

The mechanism of flocculation is mediated by the interaction between two distinct components of the cell surface: The receptors, found both on flocculent and non-flocculent cells, are α-mannan carbohydrates which bind to surface lectin-like proteins (flocculins) on adjacent flocculent cells. In this process, Ca^2+^ ions act as cofactors to maintain the active conformation of surface proteins, thereby enhancing the capacity of lectins to interact with α-mannan carbohydrates [10]. In some industries, this property has been exploited for features such as more rapid biomass recovery or clarification of fermentation products.

Many studies have shown that the *S*. *cerevisiae* lectin proteins are encoded by a family of structurally similar subtelomeric genes, *FLO1*, *FLO5*, *FLO9* and *FLO10* [11]. Another structurally similar, but non-subtelomeric gene, *MUC1/FLO11*, has been shown to be primarily responsible for several other cellular adhesion-dependent phenotypes such as pseudohyphal differentiation and invasive growth [12]. However, and when considering the large number of studies dedicated to the topic, there is little clarity about the specific role(s) of the flocculation-inducing *FLO* genes, but for the fact that they impart different levels of flocculation when expressed, are somewhat different in their ability to complement invasive growth deficiency in a *FLO11* deletion strain [13; 14], and differ in their affinity and specificity for mannobiose in *S*. *cerevisiae* [15]. The latter observation has been suggested to hold implications for self-recognition and flocculin–based social behaviour and provide some rationale for the maintenance of this multigene family [15; 16; 17].

In the wine industry, successful flocculation may support must clarification and downstream processing, limiting the need for time-consuming and expensive cell removal methods such as centrifugation and filtration. Wine yeast strains however do not display strong flocculation behaviour [18] and research has focussed on various methods to improve the flocculation of these yeasts [19; 20; 21; 22]. In naturally fermenting grape juice, *S*. *cerevisiae* wine yeast initially represent a small minority within a multispecies ecosystem consisting of dozens of other fermenting and non-fermenting yeast species, as well as bacteria and other fungi. The non-*Saccharomyces* yeasts species tend to dominate the early stages of fermentation. In the past few years, a significant research effort has focussed on the role and potential application of non-*Saccharomyces* yeasts, primarily vineyard and winery isolates, in wine production [23]. These studies have focused on the impact of these yeasts on the chemical composition of wines, including primary fermentation metabolites (i.e. glycerol and ethanol) and secondary aroma compounds which together influence the sensory characteristics and ‘quality’ of the final product [24].

However, no studies have been conducted on the flocculation behaviour and other cellular aggregation phenotypes of any of the main wine-associated non-*Saccharomyces* yeast species. Sequence data nevertheless suggest that most, if not all of these yeast species also possess lectin-encoding genes [25], suggesting that flocculation of these species may be broadly similar in nature to *S*. *cerevisiae*. A study on the flocculation mechanism of a wine-unrelated *K*. *marxianus* strain suggested that the structure and/or spatial arrangement of the cell wall groups involved in flocculation were not the same as in *S*. *cerevisiae* [26].

Here we show that many species of wine–associated non-*Saccharomyces* yeasts are able to flocculate efficiently. More interestingly, many of these species interact physically with non-flocculent wine yeast strains of *S*. *cerevisiae*, resulting in a ‘co-flocculation’ phenotype, previously only observed in the context of interactions between yeast and bacteria [27]. To further elucidate the mechanisms controlling the co-flocculation phenotypes, the role of individual *FLO* genes in such multispecies flocs was investigated. The data reveal clear differences between different *FLO* genes, with the expression of specific *FLO* genes favouring association with specific species, while excluding other species from mixed flocs. These differences between members of the *FLO* gene family suggest a wider role for these genes in controlling organisational patterns of species associations within microbial ecosystems, and provide a novel explanation for the evolutionary persistence of this large multigene family.

## Results and Discussion

### Flocculation and ‘co-flocculation’ of non-Saccharomyces isolates

**Fig 1:**
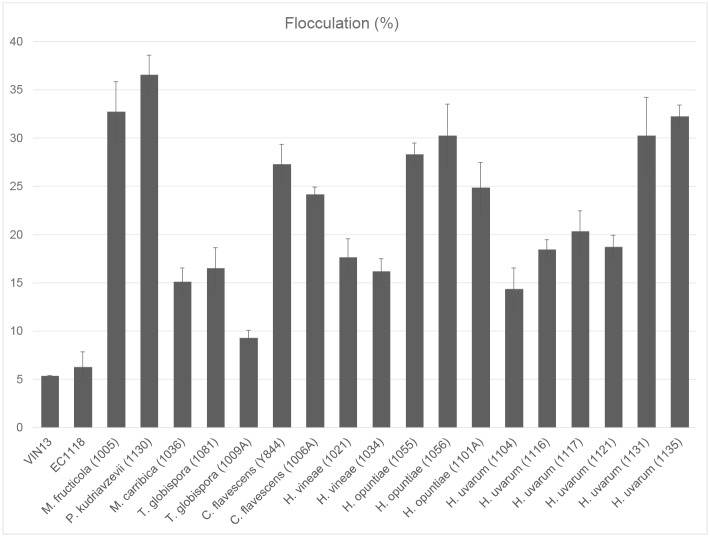
Pure culture flocculation (percentage) of the 18 non-*Saccharomyces* yeast isolates selected for this study based on their potential positive contribution to wine fermentation. Values are the average of six repeats **±** standard deviation.

Ninety-six South African isolates of wine-associated non-*Saccharomyces* yeast strains [28] were assessed for their potential usefulness in wine fermentation from an aroma production and fermentation properties perspective (data not shown). Eighteen strains showing the best fermentation potential in terms of sugar utilisation rates (and potential to decrease ethanol production) were selected (Table 1) and assessed for the extent of cell-cell aggregation and sedimentation in pure cultures in defined media. Our results indicate that most of the non-*Saccharomyces* yeasts showed higher levels of flocculation than the two control *S*. *cerevisiae* wine yeast strains VIN13 and EC1118 (Fig 1). The highest flocculation rates were seen for isolates of the species *Metchnikowia fructicola* (Y1005), *Pichia kudriavzevii* (Y1130), *Cryptococcus flavescens* (Y844 and Y1006A), *Hanseniaspora opuntiae* (Y1055 and Y1056) and *Hanseniaspora uvarum* (Y1131 and Y1135).

**Table 1:** Non-*Saccharomyces* isolates investigated in this study, as well as two *S*. *cerevisiae* controls.

Species	Strains/Isolates
*Saccharomyces cerevisiae*	VIN13, EC1118
*Pichia kudriavzevii*	IWBT-Y1130
*Metchnikowia fructicola*	IWBT-Y1005
*Meyerozyma carribica*	IWBT-Y1036
*Cryptococcus flavescens*	IWBT-Y1006A, IWBT-Y844
*Tremella globispora*	IWBT-Y1009A, IWBT-Y1081
*Hanseniaspora vineae*	IWBT-Y1021, IWBT-Y1034
*Hanseniaspora opuntiae*	IWBT-Y1055, IWBT-Y1056, IWBT-Y1101A
*Hanseniaspora uvarum*	IWBT-Y1104, IWBT-Y1116, IWBT-Y1117, IWBT-Y1121, IWBT-Y1131, IWBT-Y1135

The rate of flocculation of these isolates in the absence of Ca^2+^ was also determined (S1 Fig). All the flocculation phenotypes are calcium–dependent, the only exception being *C*. *flavescens* where a significant degree of ‘flocculation’ is still observed when Ca^2+^ is excluded from the assays, indicative of Flo–independent interactions in this species.

**Fig 2:**
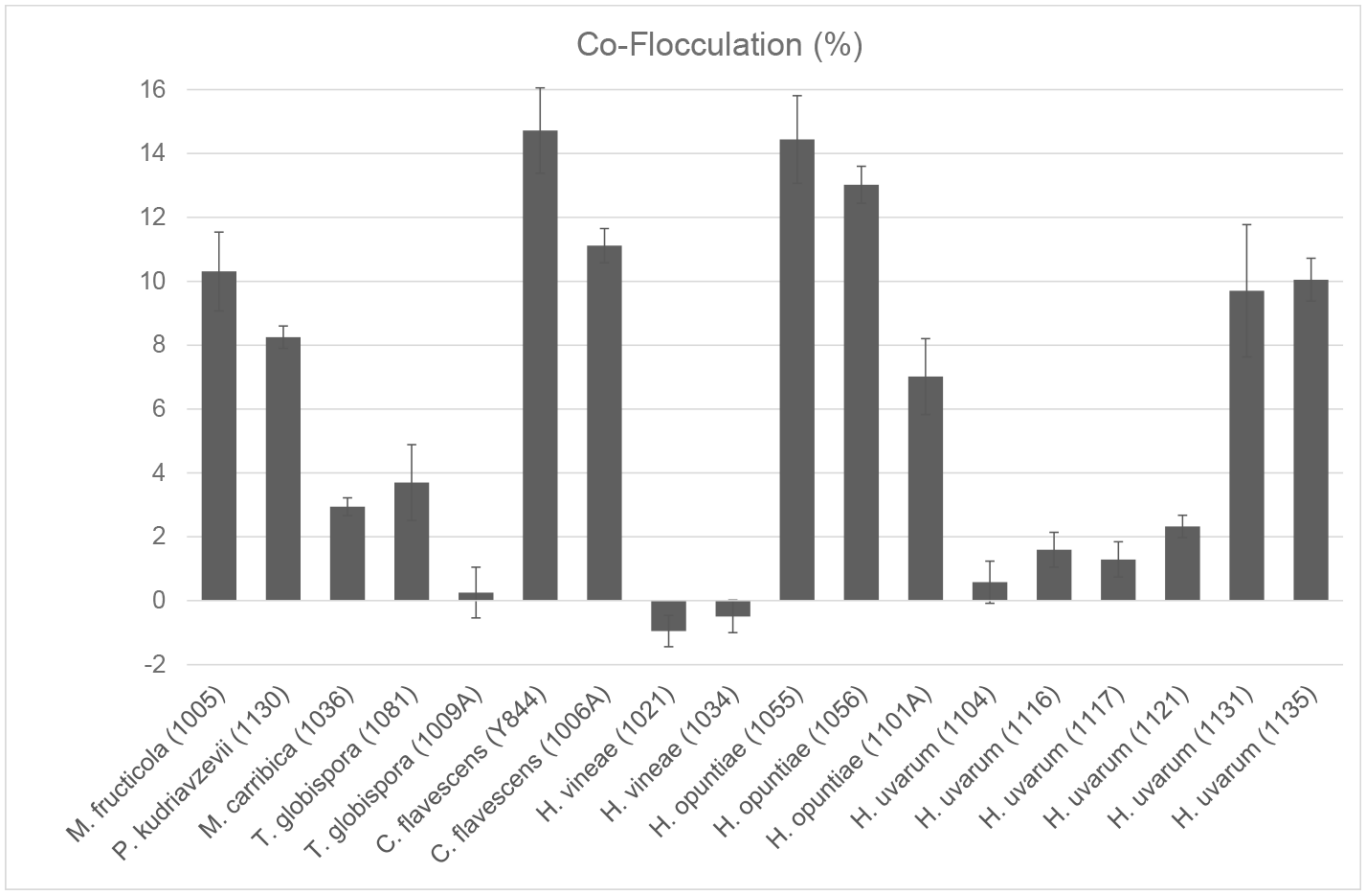
Co-flocculation (percentage) of the 18 non-*Saccharomyces* yeast isolates in combination with VIN13 (1:1 ratio of cells). Increases and decreases in flocculation percentages for the combined cultures are calculated relative to the ‘theoretical average’ flocculation based on the combined and weighted average of the corresponding pure culture flocculation percentage. Total cfu/ml were identical for pure cultures and mixed cultures (in a 1:1 ratio). Values are the average of six repeats **±** standard deviation.

Since non-*Saccharomyces* wine yeasts are increasingly being used in co-inoculation with *S*. *cerevisiae* in wine fermentation, we evaluated the flocculation behaviour in such mixed cultures (Fig 2). Flocculation assays were carried out using the non-*Saccharomyces* yeast isolates and both *S*. *cerevisiae* VIN13 and EC1118 separately (in a 1:1 ratio of non-*Saccharomyces* and *S*. *cerevisiae*). The flocculation percentages of the combined cultures were compared to those of the pure cultures. The results obtained were very similar regardless of which *S*. *cerevisiae* strain (VIN13 or EC1118) was paired with the non-*Saccharomyces* isolates for the co-flocculation assays. The degree of co-flocculation (expressed as a percentage) represents the increase in flocculation in a given mixed culture compared to the average flocculation of the two pure cultures (the data are summarised in Fig 2).

For several isolates the flocculation percentages of the combined cultures were more than 10% higher than would have been the case if the *S*. *cerevisiae* and the non-*Saccharomyces* yeasts had flocculated independently at rates observed in monocultures. Strains showing a flocculation percentages higher than approximately 20% as single cultures (Fig 1) also tended to show the greatest percentage increase (higher than 10%) in co-flocculation in combination with *S*. *cerevisiae*. Interspecies differences in co-flocculation were evident, with isolates of *C*. *flavescens* and *H*. *opuntiae* for instance showing high levels of co-flocculation compared to isolates of *H*. *vinae* and *T*. *globispora* (Fig 2).

The co-flocculation effect is highly strain-dependent and not always linked to single culture flocculation behaviour of the non-*Saccharomyces* strains. This was the case for *H*. *vineae* strains Y1021 and Y1034, where slightly negative co-flocculation values were observed even though pure cultures of these strains flocculated well on their own. A negative value for co-flocculation indicates a lower than expected flocculation value for the combined cultures, and may be indicative of a negative impact by *S*. *cerevisiae* on the ability of these species to associate, or may be due to a decrease in effective cell density and proximity between neighbouring non-*Saccharomyces* cells (total cell density remains the same for the mixed culture assays, thus the effective cell density for either of the species in the combined culture has been halved).

It is likely that the ability of the strains tested here to induce co-flocculation of VIN13 and EC1118 may show different results in combination with other wine yeast strains not considered in this study. Provided similar results are obtained under real winemaking conditions the potential to drastically improve flocculation in mixed fermentations by selecting appropriate non-*Saccharomyces* strains for co-inoculation with non-flocculent wine yeast strains may exist.

**Fig 3:**
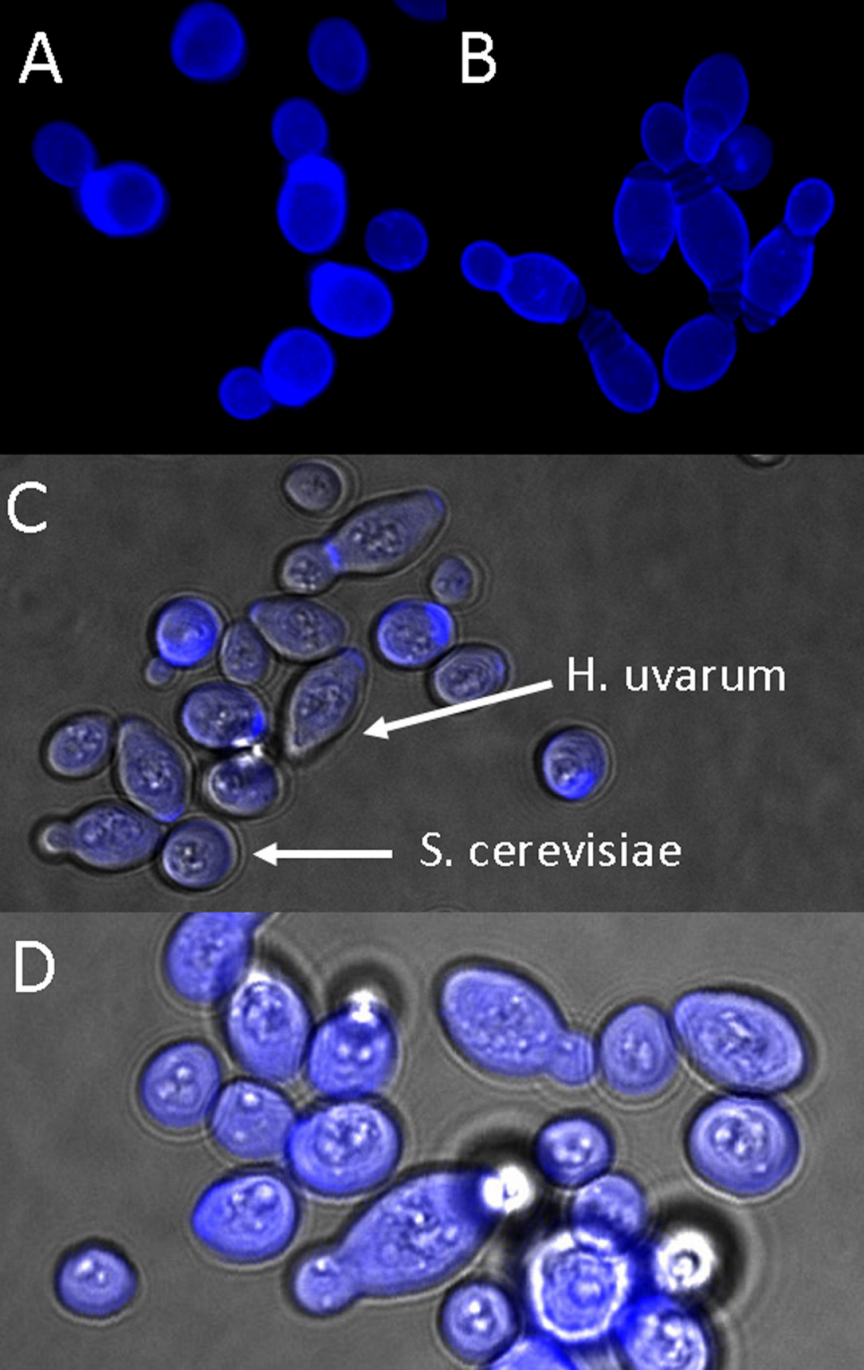
Microscopy image showing aggregation of *S*. *cerevisiae* (circular shaped cells) and *H*. *uvarum* (elongated cells) in mixed species flocs. Frames A (*S*. *cerevisiae*) and B (*H*. *uvarum*) show pure cultures of these species while frames C and D show co-aggregates of these two species.

To verify that the observed increases in flocculation were indeed due to co-flocculation of two species, microscopic imaging of sedimented cells in mixed cultures was undertaken. The data confirm the aggregation of two different species with one another in mixed species ‘flocs’ for combinations of strains that show the co-flocculation phenotype (Fig 3). Isolates of *H*. *uvarum*, *H*.*opuntiae* and *H*. *vineae* were evaluated in this regard due the morphological differences in cell shape between these cells and *S*. *cerevisiae*, allowing for simple visual discrimination.

### Genetic regulation of co-flocculation

To assess whether the co-flocculation phenotype was dependent on the presence of *FLO* genes in the *S*. *cerevisiae* strains, we investigated *FLO* gene deletion and overexpression strains in the laboratory S. cerevisiae BY4742 and FY23 genetic backgrounds. Both strains are naturally non-flocculent due to a nonsense point mutation in the *FLO8* gene [29] which encodes a transcription factor responsible for activation of key flocculin–encoding genes such as *FLO1* [30]. In our study, a strain BY4742 with restored *FLO8* activity [31] was used as the control for subsequent deletion of genes *FLO1*, *FLO10* and *FLO11* respectively (Table 2).

**Table 2:** Overexpression and deletion *S*. *cerevisiae* mutant strains.

Strain	Genotype	Reference
*Overexpression*		
FY23	MATa leu2 trp1 ura3 flo8-1	Winston et al. (1995)
FY23-F1A	MATa leu2 trp1 ura3 flo8-1 FLO1::SMR1-ADH2	Govender et al. (2008)
FY23-F1H	MATa leu2 trp1 ura3 flo8-1 FLO1::SMR1-HSP30	Govender et al. (2008)
FY23-F5A	MATa leu2 trp1 ura3 flo8-1 FLO5::SMR1-ADH2	Govender et al. (2008)
FY23-F5H	MATa leu2 trp1 ura3 flo8-1 FLO5::SMR1-HSP30	Govender et al. (2008)
FY23-F11A	MATa leu2 trp1 ura3 flo8-1 FLO11::SMR1-ADH2	Govender et al. (2008)
FY23-F11H	MATa leu2 trp1 ura3 flo8-1 FLO11::SMR1-HSP30	Govender et al. (2008)
*Deletion*		
BY4742 FLO8	MATa his3 lys2 ura3 flo8-1D::FLO8-LEU2	Bester et al. (2006)
BY4742 FLO8 flo1D	MATa his3 lys2 ura3 flo8-1D::FLO8-LEU2 flo1D::KanMX4	Bester et al. (2006)
BY4742 FLO8 flo10D	MATa his3 lys2 ura3 flo8-1D::FLO8-LEU2 flo10D::KanMX4	Bester et al. (2006)
BY4742 FLO8 flo11D	MATa his3 lys2 ura3 flo8-1D::FLO8-LEU2 flo11D::KanMX4	Bester et al. (2006)

For the overexpression of *FLO* genes, the strains described by Govender et al. [22] were used for this investigation. These strains have the native *FLO*-promoter of individual *FLO* genes replaced by either the *ADH2* or *HSP30* promoters, and allow for the assessment of the phenotypic consequences of overexpression of a particular Flo protein (Table 2). The *ADH2* promoter is subjected to carbon catabolite repression and is de-repressed with transition to growth on ethanol [32]. The *HSP30* promoter is induced during entry into the stationary phase of growth, which coincides with the depletion of glucose from the medium [33]. In addition, the *HSP30* promoter is activated by several stress factors, including heat shock and sudden exposure to ethanol. The inducible expression of three key flocculation genes (*FLO1*, *FLO5*, and *FLO11*) in the haploid laboratory strain *S*. *cerevisiae* FY23 strain thus presents a unique opportunity to compare the impact of the overexpression of individual *FLO* genes on the co-flocculation behaviour of different combinations of *S*. *cerevisiae* mutants and non-*Saccharomyces* yeasts.

**Fig 4:**
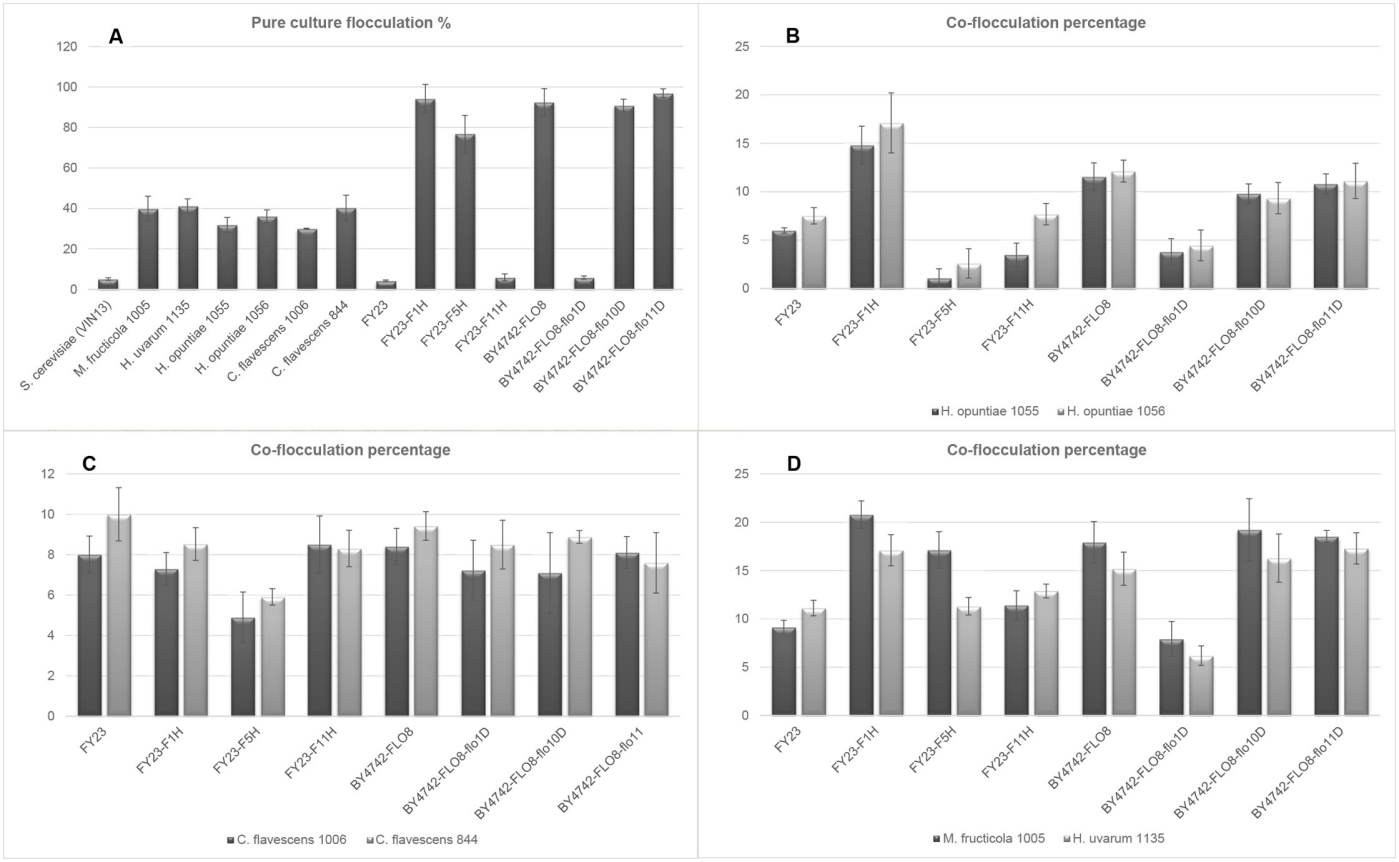
Flocculation percentage of pure culture controls of the *S*. *cerevisiae* mutant strains and six non-*Saccharomyces* isolates (frame A). Co-flocculation of mutant strains in combination with two *H*. *opuntiae* isolates (frame B), two *C*. *flavescens* (frame C) and one each of *H*. *uvarum* and *M*. *fructicola* are shown (frame D). Values are the average of six repeats **±** standard deviation.

Co-flocculation assays were carried out using the *S*. *cerevisiae* mutant strains (Table 2) in combination with a subset of six non-*Saccharomyces* isolates. The single culture flocculation percentages of the mutants and non-*Saccharomyces* isolates are shown in Fig 4A, and the co-flocculation percentages of the various combinations in Fig 4 frames B-C.

**Fig 5:**
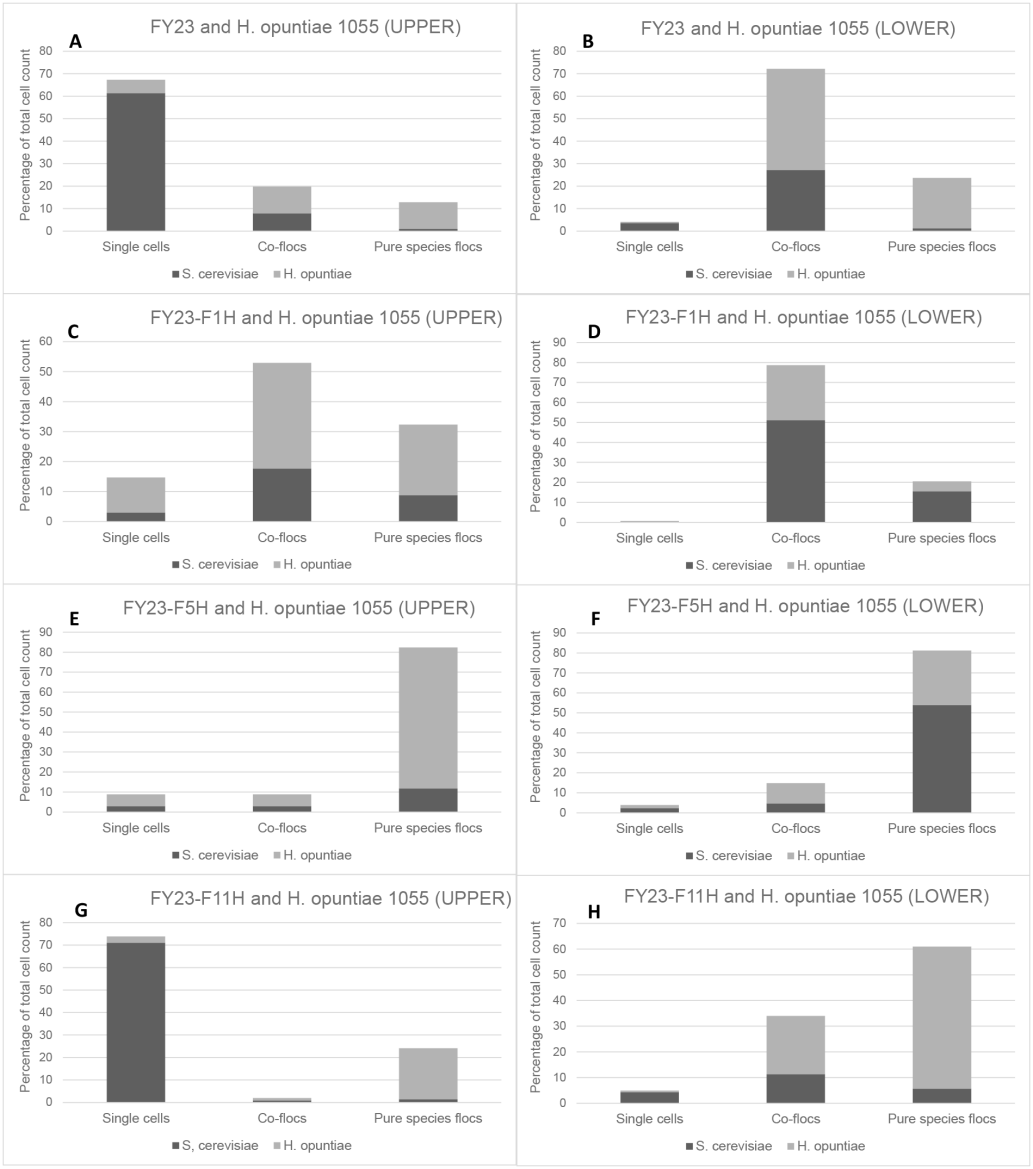
Percentage of *S*. *cerevisiae* and *H*. *opuntiae* Y1055 cells in upper non-flocculent and lower flocculent fractions, present as either, single cells, mixed species flocs, or pure species flocs. *H*. *opuntiae* in combination with FY23 is shown in frame A (upper layer) and B (lower), with FY23-FLO1 in frame C (upper) and D (lower), with FY23-FLO5 in frame E (upper) and F (lower) and in combination with FY23-FLO11 in frame G (upper) and H (lower).

Single culture fermentation percentages of mutant *S*. *cerevisiae* strains aligned with previous observations [22; 31]. Co-flocculation assays for the two species of *C*. *flavescens* (Fig 5C) show very little discernible differences in co-flocculation for any *FLO* gene deletions or overexpressions. This suggests that the co-aggregation of this species with *S*. *cerevisiae* (at least in the case of these two isolates) is not predominantly mediated by specific Flo lectin interactions, and is in line with the earlier observation that this species shows a Ca^2+^ independent flocculation behaviour (S1 Fig).

In contrast, the remaining four non-*Saccharomyces* yeasts all show *FLO* gene specific differences in co-flocculation behaviour. For instance, *FLO1* overexpression consistently leads to increased co-flocculation, while the converse is true in the case of the deletion of this gene. The importance of *FLO1* in the flocculation of *S*. *cerevisiae* has been demonstrated in numerous studies [21; 22; 30; 31]. Our data suggest that *FLO1* is likewise a key gene responsible for mediating direct cell-cell adhesion between different species of yeast in many cases.

Interestingly, *FLO5* overexpression led to a species-specific response: In combination with *M*. *fructicola* Y1005, *S*. *cerevisiae* overexpressing this gene result in an increase in co-flocculation of mixed cultures, while for *H*. *uvarum* no significant change is evident. In contrast, *H*. *opuntiae* isolates Y1055 and Y1056 both show a decrease in co-flocculation (relative to the control FY23) in co-culture with *S*. *cerevisiae* overexpressing *FLO5*. Overexpression of this flocculin thus seems to exclude the two *H*. *uvarum* isolates from physical interaction with *S*. *cerevisiae*.

Deletion of *FLO10* and *FLO11* did not impact co-flocculation behaviour in combination with any of the non-*Saccharomyces* strains tested. However, overexpression of *FLO11* led to a significant decrease in co-flocculation for this strain in combination with *H*. *opuntiae* Y1055, while not impacting the co-flocculation of *H*. *opuntiae* Y1056 compared to the control. The behaviour of the deletion and overexpression strain suggests a direct role of flocculins in terms of favouring specific associations in microbial ecosystems.

### Microscopy-based determination of mixed floc species composition

In order to further validate and investigate the impact of key genes on co-flocculation behaviour, a detailed microscopic analysis was performed on samples taken from the upper and lower layers of mixed culture co-flocculation assays. In these samples, cell counts were carried out to determine the relative number of cells in each fraction that are either in free (single cell) form, part of mixed species flocs, or part of pure (single species) flocs, for both the *S*. *cerevisiae* and non-*Saccharomyces* yeasts in co- culture. Cells in the upper layer (representative of the non-flocculating component of the culture in suspension) and lower layer (representative of the sedimented, highly flocculent component of the co-culture) were analysed separately. Cell counts were carried out in 10 randomly selected haemocytometer wells and the averages calculated (Fig 5, S2 and S3 Figs).

Clear trends are evident, corroborating the results of the co-flocculation assays (Fig 4). For the FY23 control in combination with each of the three non-*Saccharomyces* yeasts, the majority of cells in the upper fraction are single *S*. *cerevisiae* cells (Fig 5A, S2 and S3 Figs). For these strain combinations, the lower fractions show a majority representation of cells in mixed species flocs (containing more *Hanseniaspora* cells than *S*. *cerevisiae*) followed by *Hanseniaspora* in pure species flocs (Fig 5B, S2 and S3 Figs).

Data for *FLO1* overexpression also show similar trends for all three non-*Saccharomyces* isolates, with a clear majority of cells in the flocculent lower fraction (Fig 5D, S2 and S3 Figs) present in mixed species flocs, but with a majority of *S*. *cerevisiae* cells.

Interestingly, clearly different trends are evident for *FLO5* overexpression. For both *H*. *opuntiae* strains, the lower flocculent fractions contain a majority of pure species flocs, with very few mixed species flocs (Fig 5F, S2 Fig). In contrast, a significant percentage of cells in the *H*. *uvarum*–FY23-FLO5 combination are present as mixed species flocs in the lower assay fractions (S3 Fig). A comparison of putative Flo proteins has revealed that Flo5p shares 96% homology with Flo1p, making these two flocculins the most similar to one another. Since Flo5- and Flo1p play similar roles in *S*. *cerevisiae* pure culture flocculation, reasons for the maintenance of both of these genes in all known strains of *S*. *cerevisiae* has remained somewhat of an open question. Varying roles for Flo5p and Flo1p in terms of interspecies aggregation may account for, or legitimise the maintenance of these two very similar genes in the yeast genome due to possible adaptation and survival advantages in natural microbial ecosystems. In such a system, a given species of yeast could well benefit from extending social behaviour to include physical interactions with one/more other species of yeast which may collectively confer mutual benefits under fluctuating environmental conditions.

The Flo11p adhesin is the most divergent member of the family and displays only 37% similarity to Flo1p [34]. Clearly the co-flocculation outcomes of the FY23-FLO11 strain in combination with the three non-*Saccharomcyes* yeasts were very different to those obtained with either of the *FLO1* or *FLO5* overexpressing strains (Fig 5H, S2 and S3 Figs). For *H*. *opuntiae* Y1056 and *H*. *uvarum* Y1135 no differences were evident in the cell count data for these strains in combination with FY23-FLO11 compared to the corresponding control datasets. However, in the case of *H*. *opuntiae* Y1055 a decrease in mixed floc formation was evident, along with a corresponding increase in cells in pure species flocs in the lower fractions.

### Impact of *FLO* gene overexpression on co-flocculation in multispecies consortia

**Fig 6:**
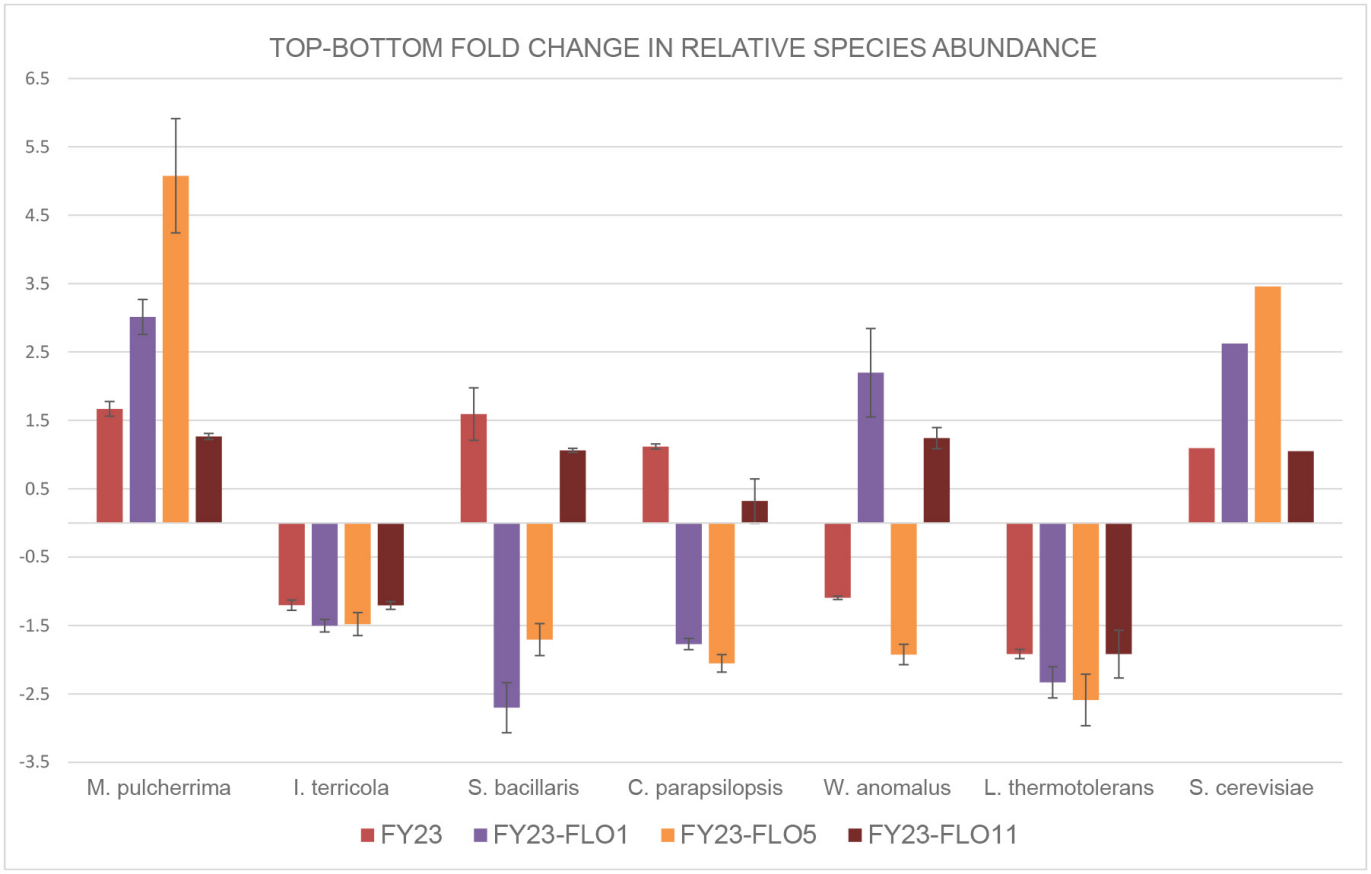
Fold change increase/decrease (based on fluorescent peak area differences) for each species between non-flocculent (top) and flocculent (bottom) fractions of assays conducted with a 6 species non-*Saccharomyces* yeast consortium in combination with the three *FLO* gene overexpressing *S*. *cerevisiae* mutant strains, as well as control FY23. The *S*. *cerevisiae* data represent the different strains used in each of the four treatments, i.e. FY32 in the case of the FY32 strain in combination with the consortium, etc. Values are the average of three biological repeats.

In order to further validate the importance of specific *FLO* genes in mediating cell-cell adhesions in mixed microbial communities, the *FLO* overexpressing strains used previously were used here in co-culture with a simulated natural wine yeast consortium. The composition of this consortium is based on natural grape must ecosystems in South Africa, and only includes species of wine-associated yeasts which have been shown to occur in high numbers in such fermentations [35]. The selective binding and floc formation of the three *FLO* overexpression strains and of the control were evaluated using automated ribosomal intergenic spacer analysis (ARISA) to calculate species abundance in total DNA extracts from the flocculation assay fractions [36]. Analysis of relative species abundance in the ‘top’ and ‘bottom’ fractions allows for the determination of ‘enriched’ species that are ‘preferred’ or rejected in terms of direct cell wall interactions with the various flocculation mutants *of S*. *cerevisiae*. In this system, it needs to be kept in mind that interactions between *S*. *cerevisiae* overexpressing flocculation genes and any one of the non-*Saccharomyces* strains may be influenced in a number of ways by the presence of the rest of the consortium. Nevertheless, the data provide a clear picture of significant differences in strain gradients in the consortium for the different treatments. Overall, the data (Fig 6) clearly demonstrate that, dependent on the expression of specific FLO genes, very different species assemblies were obtained in the flocculent, and therefore also in the non-flocculent, fraction of the culture. Some species showed significant co-enrichments, but to different degrees, while others appeared unaffected or actively excluded.

Regarding the specific impact of individual genes, FY23 and FY23-FLO11 treatments broadly result in the same outcomes for these strains of *S*. *cerevisiae* in combination with the consortium, with the exception of *W*. *anomalus* (Fig 6). For these two treatments, the fold changes in species abundance between top and bottom fractions are mostly not significantly above 1, or below -1. The exception is *Lachancea thermotolerans*, where fold changes of close to -2 are observed for all four treatments, including all three *FLO* gene overexpression mutants and the control. Likewise, a consistent (though slight) proportional decrease in *Issatchenkia terricola* was found in the bottom fractions compared to the upper fractions for all four treatments. This suggests that these two strains of non-*Saccharomyces* yeast do not sediment/flocculate well on their own, and are not interacting physically with *S*. *cerevisiae* regardless of *FLO* gene overexpression.

In contrast, the relative species abundance of *Metschnikowia pulcherrima* increased significantly from the upper to the lower flocculation fractions for all four treatments, but significantly more so in the *FLO5* and *FLO1* overexpression treatments. *FLO5* and *FLO1* thus appear to have a similar effect in terms of increasing the co-flocculation rate of this particular strain compared to other non-*Saccharomyces* strains in the consortium. *FLO1* and *FLO5* again showed similar impacts, this time in terms of a decrease in the species abundance gradient, for the strains of *Candida parapsilopsis* and *Starmarella bacillaris* (synonym, *C*. *zemplinina*). Opposite impacts were observed for *Wickerhamomyces anomalus* as *FLO1* overexpression led to an increase in the abundance of this species in the lower flocculent fractions, while *FLO5* overexpression led to a comparative decrease. Statistical analysis of relative species abundance data for the upper and lower fractions confirm the significance of these trends (S1 Table).

The results suggest the relevance of different *FLO* proteins in mediating differential interspecies aggregation in a ‘natural’ yeast ecosystem. It has been proposed that *FLO* genes are targets of strong evolutionary pressure, as yeast genomes contain a number of pseudogenes that are similar to adhesion genes, and that several DNA motifs in the central domain of these genes promote diversity of the encoded flocculins by frequent intragenic recombination events [37; 38]. It is reasonable to speculate that synergistic or antagonistic ecological interactions between different yeast species in an ecosystem may be one such selection criterion responsible for driving such adaptation of flocculin-encoding genes. In particular, given the established impact of human activity on the evolution (or domestication) of *S*. *cerevisiae* [39], the wine ecosystem may have provided an ideal environment for the evolution of such selective flocculation. It is interesting that recent data also suggest the importance of physical cell-to-cell interaction for relative survival rates of co-fermenting yeast species [40].

### Conclusions

Understanding the molecular mechanisms and regulation of flocculation in wine–relevant non-*Saccharomyces* yeasts is not only important in terms of potential industrial application, but also for our understanding of possible evolutionary mechanisms linked to physical interactions between different microorganisms in shared ecological niches. The manner in which mixed species communities control and regulate their cell-cell interactions in complex environments may provide novel insights into ecosystem evolution: Selective and specific physical associations between different species of yeast (such as those mediated by Flo proteins) are likely to be of ecological importance by allowing recruitment of compatible species and imparting growth or survival advantages. Such physical associations may increase the likelihood of metabolic exchange between cells of different species, and support the development of symbiotic associations as conditioned by the selective pressures of various natural and man-made fermentation environments.

To our knowledge this is the first report on co-flocculation behaviour in mixed cultures of *S*. *cerevisiae* and different non-*Saccharomyces* yeasts. The data suggest that adhesion phenotypes, and in particular Flo proteins, may play important roles in ecosystem dynamics—beyond the roles assigned to these properties and proteins in the past. It also suggests that the evolution of these proteins may be driven in response to specific interspecies associations within the microbial ecosystem. Further research on elucidating the molecular mechanisms underpinning the different co-flocculation behaviours of yeasts therefore will have fundamental importance for understanding ecological processes and the role of direct cell-cell interactions between different yeast species in shared environments. While the number of non-*Saccharomyces* species (and isolates of each) investigated in this study is by no means exhaustive, the data support inter- and intra-species variation in co-flocculation tendencies as well as the role of specific *FLO* genes in the regulation thereof.

Cell adhesion phenotypes are complex, and are influenced not only by genetic factors which determine the composition and properties of the yeast cell wall, but also on a number of environmental parameters which impact adhesion behaviour. The observations under controlled laboratory conditions therefore presents an over-simplified view of interspecies co-flocculation as presented here. Nonetheless, the data provide, for the first time, genetic insights into a previously unreported phenomenon.

Future studies should aim to include additional species and strains of yeast, under various experimental conditions, in order to comprehensively investigate the co-flocculation concept. Further sequencing and expression profiling of flocculation genes in non-*Saccharomyce*s yeasts would also provide an important part of the interaction picture. In addition, the relevance of yeast interspecies adhesion phenotypes to microbial interactions in natural ecosystems needs further investigation.

## Materials and Methods

### Strains, media and culture conditions

The non-*Saccharomyces* yeast strains used in the initial screen (Table 1) were selected from the strain collection of the Institute for Wine Biotechnology [28]. The wine yeast strains EC1118 and VIN13 were used as *S*. *cerevisiae* controls. The non-*Saccharomyces* isolates were characterised by RFLP analysis [41] and ribosomal RNA gene amplification and sequencing as described by Lee & Taylor [42].

The laboratory mutant strains investigated in this study are indicated in Table 2. Strains overexpressing the flocculation genes *FLO1*, *FLO5* and *FLO11* (in the FY23 genetic background) under control of two different promoter systems, namely ADH2 and HSP30, were used [22; 43]. In addition, deletion strains (BY4742 genetic background with restored *FLO8* activity) for the flocculation genes *FLO1*, *FLO10* and *FLO11* were also selected [31].

Pure freeze cultures were streaked out on YPD agar. For liquid cultures cells were cultivated in minimal media containing 0.67% yeast nitrogen base with pre-added ammonium sulfate (but without amino acids), supplemented with 2% glucose (w/v) and the required amino acids (SCD media) according to the auxotrophic growth requirements of the relevant strain. Solid medium was supplemented with 2% agar (Biolab, South Africa).

### Ca^2+^-dependent flocculation assays

Yeast colonies for each isolate were inoculated (6 repeats) in test tubes containing 5 ml SCD media and grown to stationary phase. An aqueous solution of EDTA (pH 8.0) was then added to these cultures to a final concentration of 50mM and the cultures agitated vigorously by vortexing at maximum speed setting. The OD_600_ was determined immediately by mixing 40μl of the culture with 160μl of 50mM EDTA (Reading A). Ca^2+^-dependent flocculation was then induced by spinning down 1ml of the liquid cultures in a micro centrifuge, followed by washing in 1ml ddH_2_O and re-suspension in 1ml of 40mM CaCl_2_. The samples were then vigorously agitated as before and left undisturbed for 60 seconds. A 40μl sample was taken from just below the meniscus in the micro centrifuge tube of each sample and mixed thoroughly with 160μl of a 40mM CaCl_2_ solution. A second spectrophotometric measurement was then taken at a wavelength of 600nm as before (Reading B). For more information see Bester *et al*. [31]. The extent of Ca^2+^-dependent flocculation was then calculated using the following formula:

$$Flocculation\;\left(\%\right)\;=\;\frac{A-B}{A}\times \;100$$

To calculate the extent of co-flocculation, *S*. *cerevisiae* (VIN13 and EC1118) and the non-*Saccharomyces* yeast under investigation were combined in a 1:1 cell:cell ratio and the flocculation assay carried using the mixed culture as described in the preceding section. The total cell concentrations in the co-flocculation assays (i.e. *S*. *cerevisiae* plus non-*Saccharomyces* strain) were the same as for pure cultures. The flocculation percentage is calculated as before, and the ‘co-flocculation’ percentage is calculated by subtracting the ‘expected’ flocculation (based on the combined average flocculation percentage of the pure cultures) from the experimentally determined flocculation percentage obtained for the combined cultures. Therefore;

Co_flocculation (%) = experimentally determined flocculation of mixed culture (%)– expected flocculation (%)

Similarly, co-flocculation percentages were determined for strains Y1005, Y1006, Y1055, Y1056, Y1130, Y1135 and Y844, each in combination with the 11 *S*. *cerevisiae* mutant strains listed in Table 2. The three HSP30 inducible overexpression constructs were subject to 45 mins heat shock at 42°C as described by Govender et al. [20] in order to activate gene overexpression before flocculation assays were carried out. Pure culture and co-flocculation assays were conducted with six biological repeats of each.

### Microscopy

Calcofluor white (Fluka Analytical, Sigma-Aldrich) staining of cells and fluorescence microscopy was carried out as described by de Groot et al. [44]. Image acquisition was performed on an Olympus Cell system attached to an IX 81 inverted fluorescence microscope equipped with an F-view-II cooled CCD camera (Soft Imaging Systems). The excitation laser used was the violet laser with 407 nm wavelength and the emission filter used was the Pacific Blue channel with a 450/40 band pass filter. Images were processed and background-subtracted using the Cell software, and presented in a maximum intensity projection.

Co-flocculation behaviour of *H*. *opuntiae* isolates Y1055 and Y1056, and *H*. *uvarum* isolate Y1135 were further investigated in combination with three *S*. *cerevisiae FLO* gene overexpression mutants (FY23-F1H, FY23-F5H, FY23-F11H) and the control FY23. Cell cultures were combined in a 1:1 ratio of the non-*Saccharomyces* yeast under investigation in combination with each of the *FLO* gene overexpressing strains and control FY32 separately (1 x 10^7^ cells/ml of each). Flocculation assays were conducted as described previously, and 50 ul samples were taken (after a 1 min flocculation time) from the upper phase (top) of the liquid cultures (enriched for the non-flocculent component of the mixed culture) as well as at the bottom of the liquid cultures (enriched in sedimented flocs).

These two fractions from each flocculation assay were subjected to microscopic analysis on the Olympus Cell^R system (Olympus Biosystems, GMBH). Cells were visualised (100X objective) using a haemocytometer (Spencer, AO instruments) and image capture was performed using the F-view-II cooled CCD camera (Soft Imaging systems). The haemocytometer counting grid (200 μM diameter wells used) allowed for the differential enumeration of *S*. *cerevisiae* and species from the genus *Hanseniaspora* (by eye, based on cell morphology) in the different fractions. Total cell counts for both the *S*. *cerevisiae* and *Hanseniaspora* species were determined in ‘top’ and ‘bottom’ fractions, both in free form and as part of multicellular flocs, in 10 grids for each sample, and the averages calculated. Values are presented as the percentage of each species in free or floc form in the different fractions.

### Automated ribosomal intergenic spacer analysis

A yeast consortium consisting of 6 non-*Saccharomyces* species, similar to those found in a natural fermenting must at the start of fermentation, was established consisting of *M*. *pulcherrima*, *I*. *terricola*, *S*. *bacillaris*, *C*. *parapsilopsis*, *W*. *anomalus* and *L*. *thermotolerans* strains isolated from South African vineyards. Overnight cultures of these strains were grown individually in YPD and pooled to form a mixed culture. This mixed culture yeast consortium was used to conduct co-flocculation assays in combination with three *S*. *cerevisiae FLO* gene overexpression mutants (FY23-F1H, FY23-F5H, FY23-F11H) and the control FY23. Samples were once again taken from the top and bottom fractions of the assay tubes as representative of the non-flocculent and flocculent cells respectively. DNA extraction was carried out on these samples as described by Hoffman [45]. ARISA analysis was subsequently performed (using 50ng of DNA template) and caboxy-fluorescein labelled forward (ITS1-6FAM) and ITS4 primers [36; 46]. The labelled PCR products were separated by capillary electrophoresis on an ABI 3010×I Genetic analyzer (Applied Biosystems) at the Central Analytical Facility, Stellenbosch University. The raw data were converted to electropherograms and further analysed in Genemapper 4.1 (Applied Biosystems). Peak areas for each species in the consortium as well as *S*. *cerevisiae* were calculated to determine the relative species abundance in each fraction. The average abundance of each of the individual peaks was calculated and represented as a percentage of the total number of peak heights displayed in each sample.

### Statistical analysis

T-tests and ANOVA were conducted using Statistica (version 10.2).

## Supporting Information

S1 Fig Calcium-independent pure culture sedimentation rates (percentage) of the 18 non-*Saccharomyces* yeast strains investigated. Values are the average of six repeats **±** standard deviation.
(TIF)

S2 Fig Percentage of *S*. *cerevisiae* and *H*. *opuntiae* 1056 cells in upper non-flocculent and lower flocculent fractions, present as either single cells, mixed species flocs, or pure species flocs. *H*. *opuntiae* in combination with FY23 is shown in frame A (upper layer) and B (lower), with FY23-FLO1 in frame C (upper) and D (lower), with FY23-FLO5 in frame E (upper) and F (lower) and in combination with FY23-FLO11 in frame G (upper) and H (lower).
(TIF)

S3 Fig Percentage of *S*. *cerevisiae* and *H*. *uvarum* 1135 cells in upper non-flocculent and lower flocculent fractions, present as either single cells, mixed species flocs, or pure species flocs. *H*. *uvarum* in combination with FY23 is shown in frame A (upper layer) and B (lower), with FY23-FLO1 in frame C (upper) and D (lower), with FY23-FLO5 in frame E (upper) and F (lower) and in combination with FY23-FLO11 in frame G (upper) and H (lower).
(TIF)

S1 Table Summary of the ANOVA analysis of ARISA data for non-flocculent upper and flocculent lower culture fractions (p<0.05) of the yeast consortium in combination with four different *S*. *cerevisiae* strains (FY32, FY32-FLO1, FY32-FLO5, FY32-FLO11). Lowercase letters are used to discriminate between species which are statistically significantly different in their abundance between assays conducted with the three different flocculation mutant strains and control.
(DOCX)
