# Supplementary figures and images for: Co-Flocculation of Yeast Species, a New Mechanism to Govern Population Dynamics in Microbial Ecosystems
Source: PLoS One. 2015 Aug 28;10(8):e0136249. doi: 10.1371/journal.pone.0136249 (PMC4552943; doi:10.1371/journal.pone.0136249)

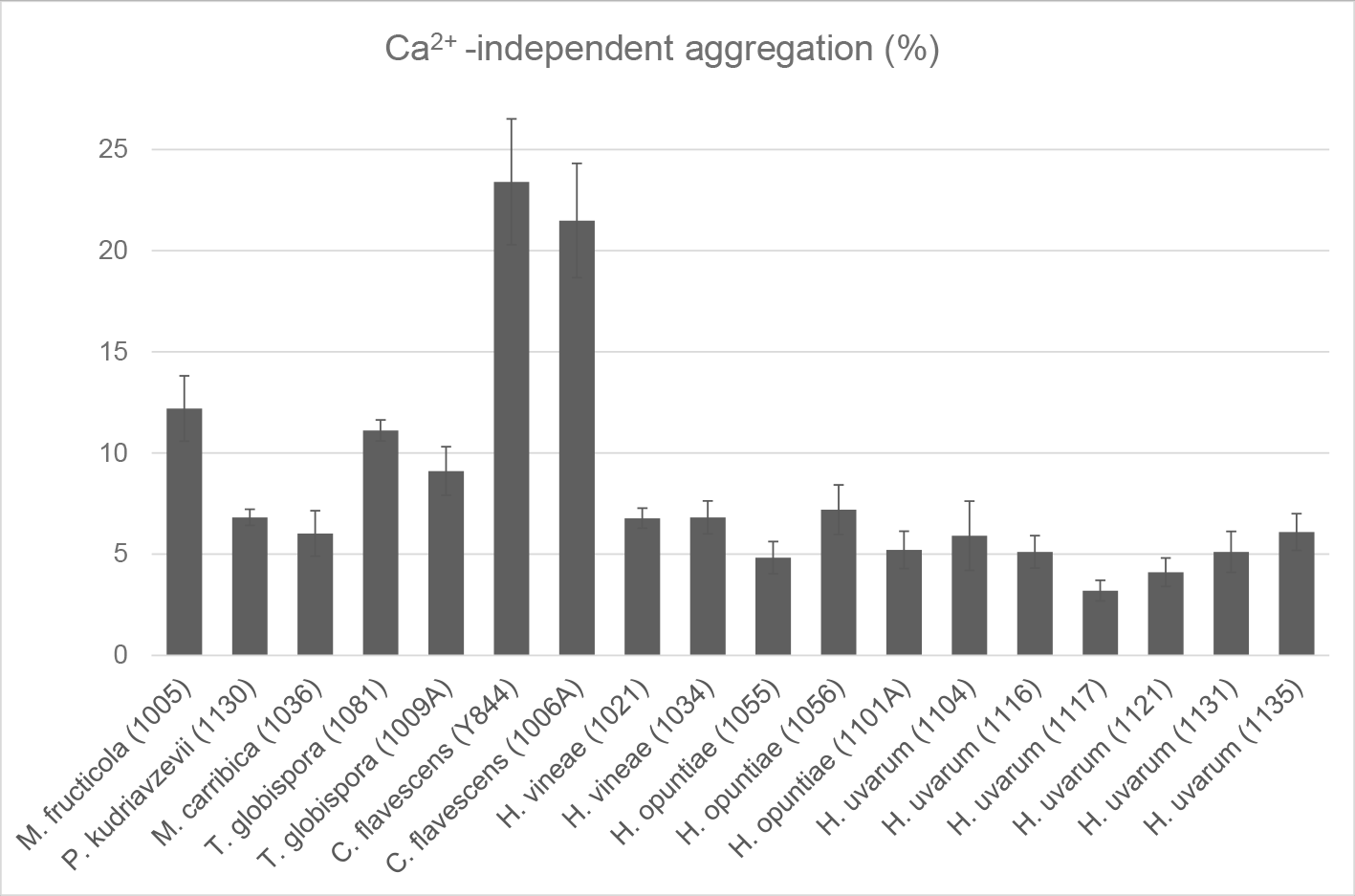

Supplement: S1 Fig — Values are the average of six repeats ± standard deviation. (TIF) [file pone.0136249.s001.tif]

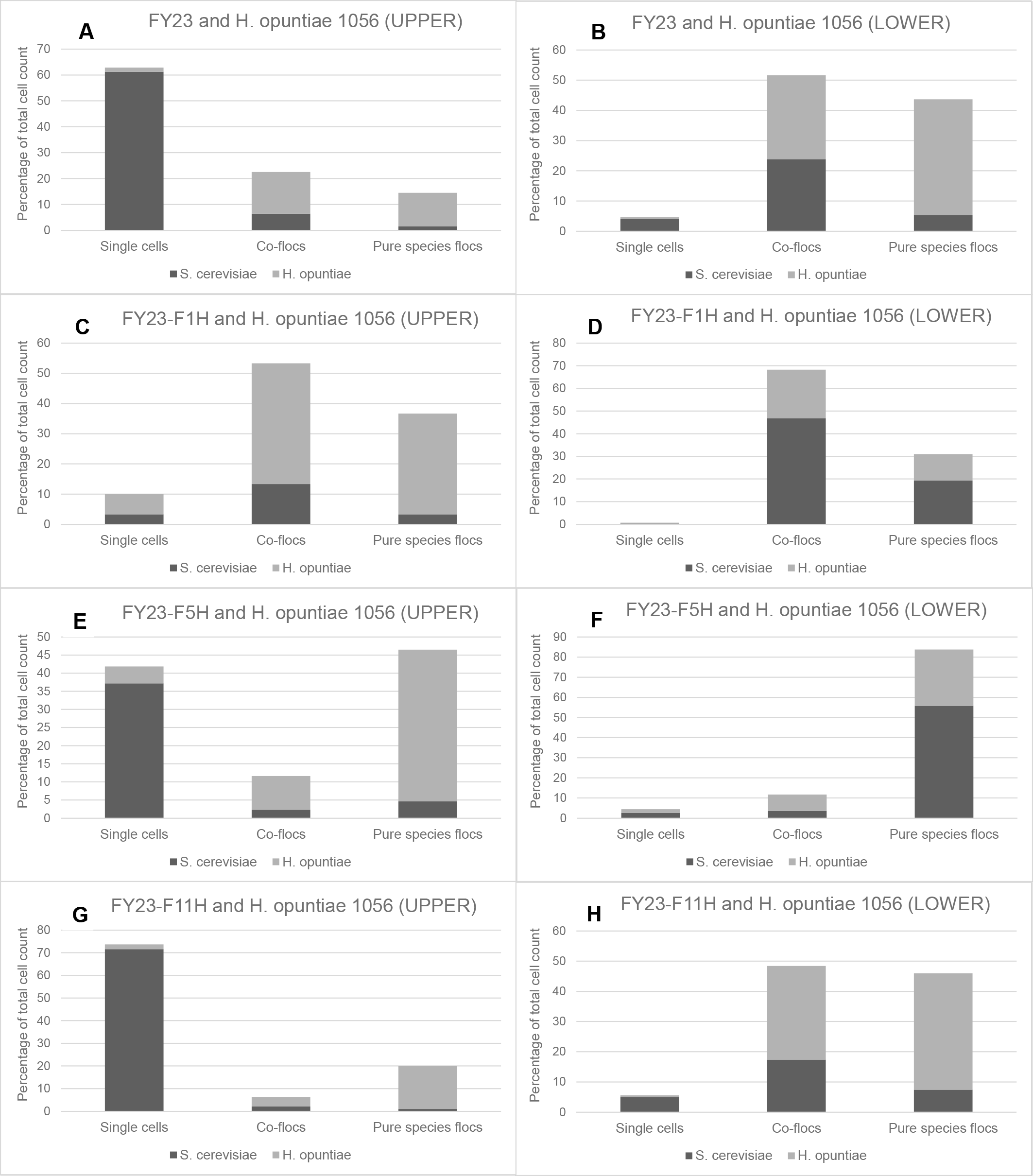

Supplement: S2 Fig — H. opuntiae in combination with FY23 is shown in frame A (upper layer) and B (lower), with FY23-FLO1 in frame C (upper) and D (lower), with FY23-FLO5 in frame E (upper) and F (lower) and in combination with FY23-FLO11 in frame G (upper) and H (lower). (TIF) [file pone.0136249.s002.tif]

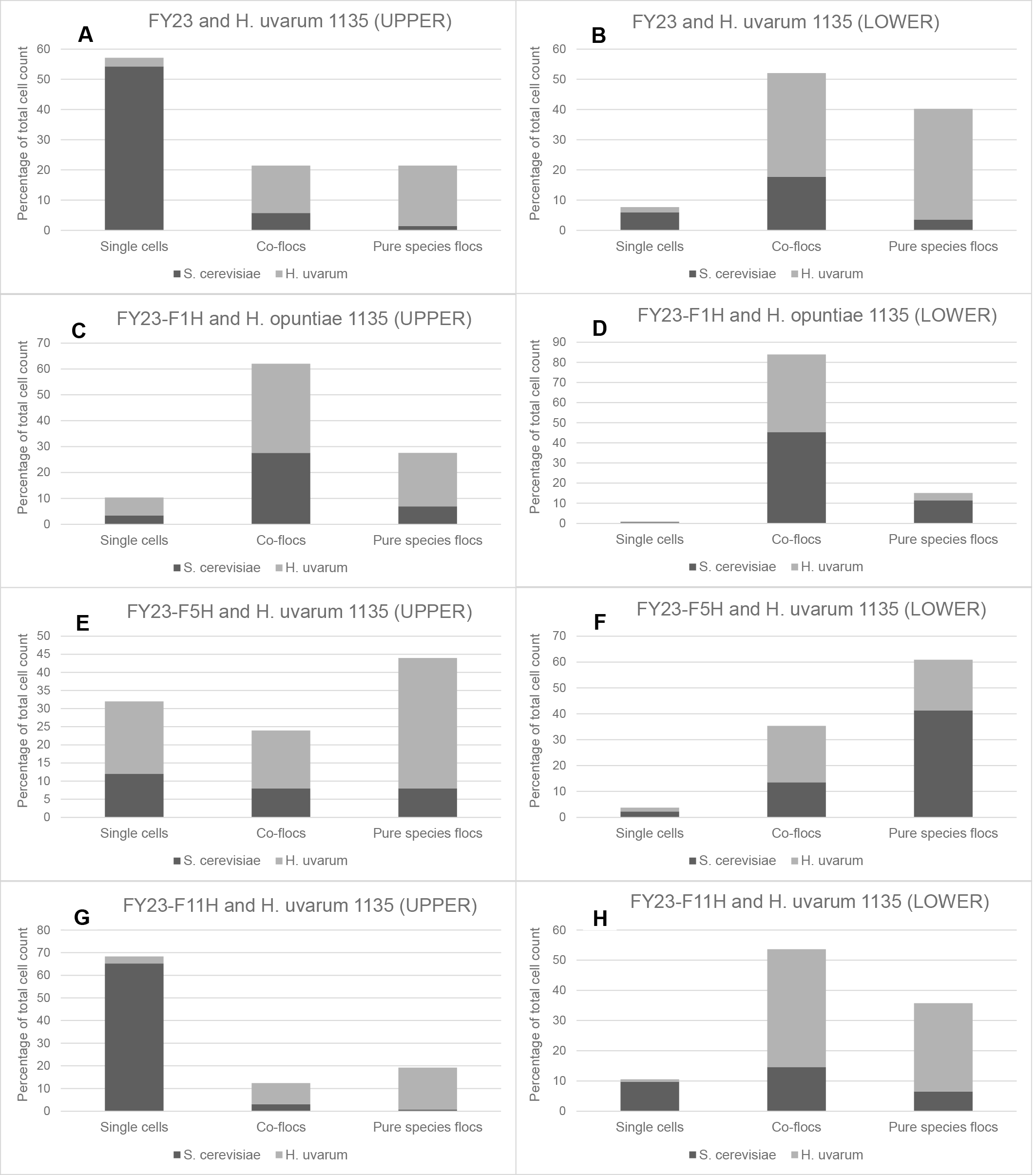

Supplement: S3 Fig — H. uvarum in combination with FY23 is shown in frame A (upper layer) and B (lower), with FY23-FLO1 in frame C (upper) and D (lower), with FY23-FLO5 in frame E (upper) and F (lower) and in combination with FY23-FLO11 in frame G (upper) and H (lower). (TIF) [file pone.0136249.s003.tif]
