# Supplementary material for: Co-Flocculation of Yeast Species, a New Mechanism to Govern Population Dynamics in Microbial Ecosystems
Source: PLoS One. 2015 Aug 28;10(8):e0136249. doi: 10.1371/journal.pone.0136249 (PMC4552943; doi:10.1371/journal.pone.0136249)
Supplement: S1 Table — Lowercase letters are used to discriminate between species which are statistically significantly different in their abundance between assays conducted with the three different flocculation mutant strains and control. (DOCX) [file pone.0136249.s004.docx]

| YEAST SPECIES | TREATMENT | |  |  |
| --- | --- | --- | --- | --- |
| Upper fraction: | FY32 | FLO1 | FLO5 | FLO11 |
| M. pulcherrima | a | b | b | a |
| I. terricola | a | a | b | a |
| S. bacillaris | a | b | c | a |
| C. parapsilopsis | a | b | c | a |
| W. anomalus | ab | c | b | a |
| L. thermotolerans | a | b | b | a |
| S. cerevisiae | a | b | b | a |
| Lower fraction: | FY32 | FLO1 | FLO5 | FLO11 |
| M. pulcherrima | a | a | c | b |
| I. terricola | ab | a | c | b |
| S. bacillaris* | a | a | a | a |
| C. parapsilopsis | a | b | a | a |
| W. anomalus | ac | b | ab | c |
| L. thermotolerans | a | bc | ab | c |
| S. cerevisiae | a | b | ab | ab |
